# Effects of Excessive Alcohol Use on Antisocial Behavior Across Adolescence and Early Adulthood

**DOI:** 10.1016/j.jaac.2017.07.781

**Published:** 2017-10

**Authors:** Gemma Hammerton, Liam Mahedy, Joseph Murray, Barbara Maughan, Alexis C. Edwards, Kenneth S. Kendler, Matthew Hickman, Jon Heron

**Affiliations:** a Population Health Sciences, University of Bristol, Bristol, UK; b Postgraduate Program in Epidemiology, Universidade Federal de Pelotas, Pelotas, Brazil; c Institute of Psychiatry, Psychology and Neuroscience, King’s College London, London, UK; d Virginia Institute for Psychiatric and Behavioral Genetics, Virginia Commonwealth University, Richmond

**Keywords:** Avon Longitudinal Study of Parents and Children, alcohol consumption, antisocial behavior, within-person effect, between-person effect

## Abstract

**Objective:**

Antisocial behavior (ASB) decreases with age in most of the population; however, excessive alcohol use can inhibit the desistance process. This study investigated whether excessive early drinking might slow a young person’s overall pattern of crime desistance compared with that of others (“between-person effects”) and whether short-term increases in alcohol consumption might result in short-term increases in ASB (“within-person effects”).

**Method:**

Frequency of ASB and typical alcohol consumption were assessed repeatedly in young people 15 to 21 years old in a population-based birth cohort (Avon Longitudinal Study of Parents and Children). Longitudinal trajectories showed ASB decreasing and alcohol use increasing across adolescence, which stabilized in adulthood. The parallel growth model was re-parameterized to simultaneously estimate the person-specific (or “between-person”) and time-specific (or “within-person”) influences of alcohol on ASB.

**Results:**

Typical alcohol consumption by young people 15 years old was positively associated with ASB cross-sectionally and into young adulthood (i.e., there were between-person effects of initial levels of alcohol consumption on initial [b 1.64, standard error 0.21; *p* < .001] and final [b 0.53, standard error 0.14; *p* < .001] levels of ASB). Within-person effects also were identified in early adulthood (b 0.06, standard error 0.02; *p* = .001), showing that when a young person reported consuming more alcohol than normal across the past year, that person also reported engaging in higher than usual levels of ASB.

**Conclusion:**

The results are consistent with between- and within-person effects of excessive alcohol use on ASB desistence. Future research should further investigate this relation by investigating pathways into excessive alcohol use and ASB in adolescence.

Antisocial behavior (ASB) is a major public policy and health concern^1^; it not only places a large financial burden on society^1^, ^2^ but also is associated with increased risk of negative outcomes, including criminal behavior^3^, ^4^ and mental health disorders.^4^ In addition to prevention strategies before ASB onset, key targets for intervention can arise later in development. For example, the age–crime curve consistently shows that ASB peaks in mid-adolescence and then decreases throughout late adolescence and early adulthood.^3^, ^4^ However, there is evidence for individual differences in the course of ASB across this period,^4^ and identifying factors associated with desistance is important to guide post-onset interventions.^5^

There are ways in which an exposure such as excessive alcohol use might promote ASB, limiting the decrease typically seen through late adolescence.^4^, ^6^ First, excessive alcohol use can slow a young person’s overall pattern of crime desistance compared with that of others (known as “between-person effects”). Between-person effects provide evidence for who is at risk and can be tested with covariates that are present before or when ASB begins to decrease. Support for this hypothesis has mainly come from studies examining between-person differences in the long-term course of ASB predicted by baseline levels of alcohol use.^6^, ^7^, ^8^ Results generally show that baseline alcohol use is associated with ASB cross-sectionally; however, findings regarding the effect of alcohol on ASB desistance have been more mixed.^6^, ^7^, ^8^, ^9^ Second, alcohol use can affect desistance from ASB through a series of short-term, time-specific influences^6^, ^7^, ^8^, ^10^, ^11^ (known as “within-person effects”). In contrast to between-person effects, within-person effects focus on when a person is at risk.^6^

In the present study, we aim to contribute to the literature in 2 ways. First, few studies have examined these person-specific (or between-person) and time-specific (or within-person) influences in the same model. Recent work by Curran *et al.*^12^, ^13^, ^14^ has detailed advances in analytical approaches that allow the between- and within-person effects to be disaggregated. In addition, prior studies that have examined within-person effects have generally treated alcohol use as a time-varying covariate, rather than modeling a longitudinal trajectory; therefore, the time-varying measurements of alcohol confound variance from adolescents’ typical trajectory versus that from time-specific deflations in that trajectory. Modeling the characteristic (and differing) trajectories for ASB and alcohol use across adolescence is important not only to obtain reliable estimates for the within- and between-person effects^12^ but also to allow the effect of drinking more alcohol than usual on short-term increases in ASB to be examined. Second, findings to date are mainly based on a selection of small, specific samples, such as male offenders,^7^ those in treatment for substance use,^11^ and single-sex samples^6^, ^8^, ^10^, ^15^ or focus on specific outcomes such as dating aggression^16^ and psychopathic features^17^ rather than ASB more generally.

The present investigation expands on the extant literature in its use of a large, prospective population cohort to examine whether excessive alcohol use acts as a snare and decreases the rate of decline in ASB across young adulthood. The specific aims are to examine changing patterns of typical alcohol consumption and ASB in tandem across adolescence and early adulthood, examine the between-person effects of typical alcohol consumption in mid-adolescence on the course of ASB into young adulthood, and investigate the within-person, time-specific effects of alcohol consumption on ASB. The hypothesis was there would be between- and within-person effects of alcohol consumption on the desistence of ASB.

## Method

### Sample

Data were used from a large UK birth cohort, the Avon Longitudinal Study of Parents and Children (ALSPAC), which was set up to examine genetic and environmental determinants of health and development.^18^ The “core” enrolled sample consisted of 14,541 pregnant women residing in the former county of Avon in the United Kingdom who had an expected date of delivery from April 1, 1991 to December 31, 1992. Of the 13,988 offspring alive at 1 year of age, a small number of participants withdrew consent (n = 24). The sample also was restricted to singletons or first-born twins, leaving a starting sample of 13,775. Parents and children have been followed up regularly since recruitment by questionnaire and clinic assessments. Further details on the sample characteristics and methodology have been described previously.^18^, ^19^ Detailed information about the ALSPAC and a data dictionary can be found at the study website (http://www.bristol.ac.uk/alspac) and at http://www.bris.ac.uk/alspac/researchers/data-access/data-dictionary. Ethical approval for the study was obtained from the ALSPAC ethics and law committee and the local research ethics committees.

### Measures

A timeline for data collection is shown in Figure S1 (available online).

#### Antisocial Behavior

A self-report questionnaire asking about antisocial acts committed in the past year^20^ was completed by the young person at 4 time points from 15 to 21 years of age. At approximately 15 years (mean 15 years 6 months, standard deviation [SD] 4 months) and approximately 18 years of age (mean 17 years 10 months, SD 5 months), data were collected during a computer-based session at a focus clinic; at approximately 19 years (mean 18 years 8 months, SD 6 months) and 21 years of age (mean 20 years 11 months, SD 6 months), data were collected by online or postal questionnaire. Eight ASB items were consistent across all time points (stole from shops, broke into a vehicle or building, stole from a person, damaged property, assault, carried a weapon, rowdy in a public place, hurt animals). For each item, respondents were asked, “How often in the last year have you …,” with responses classified into 3 categories: “not at all,” “just once,” and “2 or more times.” Then, all items were combined to create a sum score representing the frequency of antisocial acts committed in the past year at each time point (range 0–16). External validity for this self-report questionnaire has been examined previously using cross-checks with agency records and teachers’ questionnaires.^21^

#### Alcohol Consumption

At each time point (15, 18, 19, and 21 years) respondents were asked to report the number of units of alcohol they consumed daily when they had a drink during the past year, with responses classified into 5 categories: “none” (score 0), “1 or 2” (score 1), “3 or 4” (score 2), “5 or 6” (score 3), “7 to 9” (score 4), and “10 of more” (score 5). Sensitivity analyses were performed using a measure of alcohol frequency. Respondents were asked to report how often in the past year they had a drink containing alcohol, with responses classified into 4 categories: “never,” “occasional,” “weekly,” and “daily or almost daily.”

#### Covariates

Maternal questionnaires completed during pregnancy were used to assess housing tenure (owned or mortgaged; privately rented; subsidized housing rented from council or housing association), maternal level of education (no high school qualifications; high school only; beyond high school), parity (study child 1, 2, 3, or subsequent child born in family), and crowding (up to 1 person per room in house; >1 person per room). These sociodemographic variables were included in all analyses primarily to aid in addressing potential bias from missing data; however, they also could be confounders of the between-person effect of alcohol consumption on ASB.

Developmental trajectories of conduct problems at 4 to 13 years old, exposure to antisocial peers at approximately 11 years old, and parental crime and problematic alcohol use from the child’s birth to 11 years also were included as potential confounders in secondary analyses. Details of the assessment of these confounders are provided in Supplement 1 (available online).

### Data Analysis

#### Parallel Growth Model for Typical Alcohol Consumption and ASB

**Figure 1:**
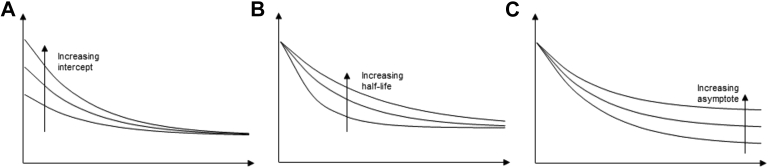
Example of exponential decay trajectories showing impact of changes in growth factors. Note: Panel A shows the impact of changing the intercept with the half-life and asymptote held constant. Panel B shows the impact of changing the half-life with the intercept and asymptote held constant. Panel C shows the impact of changing the asymptote with the intercept and half-life held constant.

Longitudinal trajectories for typical alcohol consumption and ASB were derived using a parallel exponential growth model. These growth curves were specifically selected based on a combination of exploring the shape of the population mean change for the 2 constructs and selecting a theoretically justifiable functional form (Supplement 2, available online). In the traditional exponential growth model, 3 growth factors are estimated: intercept, rate, and asymptote. The intercept (when fixed at baseline) is the average predicted starting point or initial level, and the asymptote is a line that the curve approaches as it heads towards infinity, or the average predicted final level. The rate represents the manner in which the asymptote is approached. In the present study, the model was re-parameterized to estimate the “half-life” instead of the rate. The half-life, measured in years, is the time by which 50% of a person’s total change has been observed. Therefore, it is not only more interpretable than the rate but also can be easily compared across measures with different scales. In addition, the half-life is of greater interest for examining desistance from ASB, because it provides an indication of the time taken for a person to desist. In an exponential growth model, the factor loadings are a function of the estimable parameters, and the loading for the final repeated observed measure on the asymptote indicates the total change to the asymptotic level that is gained by the end of the study.^22^ This factor loading is important to consider because extrapolation far beyond the period of observation can result in an asymptote that is poorly estimated. Figure 1 shows example estimated exponential decay trajectories that differ on the intercept (Figure 1A), the half-life (Figure 1B), and the asymptote (Figure 1C). Further information on the exponential model is presented in Supplement 2 (available online).

#### Addressing Age Variability

Age variability within a wave is common in longitudinal studies, and there is a range of methods for incorporating this age variation into the growth model. In the present study, we opted to preserve some, but not all, age variation by dividing respondents at each wave into younger and older groups and treating these groups as 2 separate time points in the trajectory analyses. Therefore, a total of 8 time points of data were analyzed; however, each respondent contributed only a maximum of 4 pieces of information, akin to an accelerated design. The mean ages at each time point were 15 years 3 months, 15 years 8 months, 17 years 6 months, 18 years 1 month, 18 years 4 months, 19 years 1 month, 20 years 6 months, and 21 years 4 months.

#### Between-Person Effects

An unconditional parallel exponential growth model for ASB and typical alcohol consumption was estimated. There are different between-person effects that can be estimated with a parallel growth model; however, the focus was the effect of baseline alcohol use on the ASB trajectory. Therefore, the ASB growth factors (intercept, half-life, and asymptote) were regressed on the latent intercept for alcohol consumption. Then, the model was re-parameterized to examine the effect of the alcohol intercept on the ASB growth factors, after accounting for the effect of the ASB intercept on the ASB half-life and asymptote. This was necessary because the relation between initial alcohol levels and the rate of decrease in ASB is likely to be dependent on a person’s initial ASB level. In addition, although initial alcohol levels affect final ASB levels directly and indirectly, through the effect on initial ASB levels, it is the direct effect that is most relevant for identifying factors related to ASB desistance.

#### Within-Person Effects

**Figure 2:**
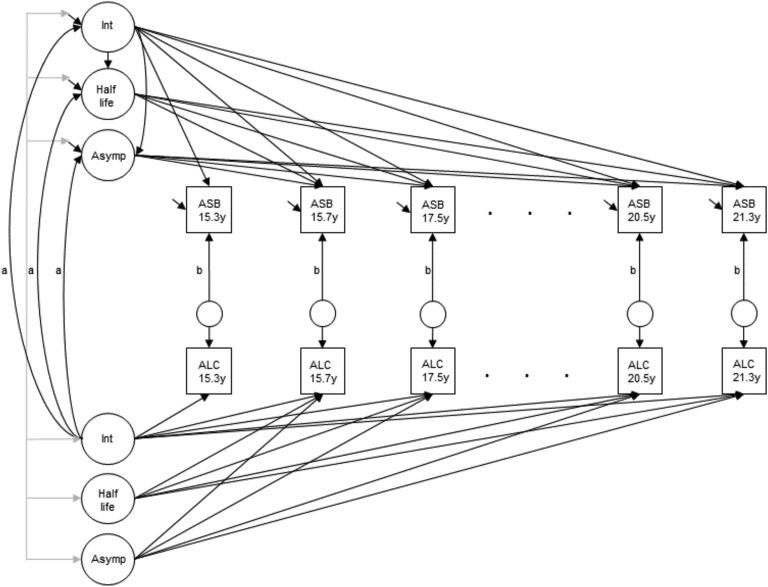
Parallel exponential growth model for antisocial behavior (ASB) and typical alcohol consumption (ALC) showing between-person effects of alcohol intercept on ASB growth factors and within-person effects of repeated typical alcohol consumption on repeated ASB. Note: a = paths testing between-person effects; Asymp = asymptote; b = paths testing within-person effects; Int = intercept.

Within-person effects of typical alcohol consumption on ASB were examined by regressing the observed repeated ASB measure (net the underlying ASB trajectory) on the time-specific residual for repeated alcohol consumption. The time-specific residual represents the deviation between the observed repeated alcohol measure and the underlying alcohol trajectory (Figure S2, available online). If the repeated ASB measures are regressed directly on the observed alcohol measures, instead of on the alcohol residuals, the observed alcohol measures serve as mediators for the influence of the alcohol growth factors on the repeated ASB measures, meaning that the between-person effects will be altered with the inclusion of the within-person effects. By specifying the time-specific residuals for alcohol use and using these residuals in the time-specific regressions, this mediated pathway is interrupted, thereby allowing the between- and within-person effects to be estimated simultaneously.^14^ The repeated ASB measure was regressed on the time-specific residual for alcohol at the same assessment, because it was hypothesized that alcohol consumption would have a proximal rather than a lagged effect on ASB as has been found previously.^6^ The final model is shown in Figure 2.

#### Model Fit

In the parallel growth model, an additional parameter was included to allow the trajectory functions to absorb artifactual differences between clinic and questionnaire data collection that might be due to the respondents’ tendency to more readily report antisocial acts or alcohol use with the privacy of a questionnaire assessment completed at home. We note that this is equivalent to including an assessment-technique (clinic or questionnaire) dummy variable as a fixed effect in the multilevel modeling formulation of a latent growth model. The time-specific residual (co)variances for repeated measures (and therefore the within-person effects) also were permitted to be heteroskedastic between but not within assessment technique. Subsequently, fit for each trajectory was evaluated by examining residuals for the mean and covariance structure, and the final model fit was examined using model fit statistics (root mean square error of approximation and comparative fit index). Root mean square error of approximation values below 0.05 and comparative fit index values above 0.90 indicate close fit. All models were analyzed in Mplus 7.4 using maximum likelihood estimation with robust standard errors (SEs).^23^ An annotated Mplus script for the final analysis model is available on request.

#### Missing Data

Missing data were handled using full information maximum likelihood. Full information maximum likelihood makes the assumption that data are missing at random (i.e., given the observed data included in the model, the missing-ness mechanism does not depend on the unobserved data). This assumption was made more plausible by the inclusion of a number of auxiliary variables related to missing data. Young people who had complete data for ASB and typical alcohol consumption from at least 1 of the 4 time points were included in the trajectory analyses (n = 6,699). The inclusion of sociodemographic confounders resulted in a sample size 6,112 (2,772 male, 3,340 female). Figure S3 (available online) shows a flowchart of retention in the ALSPAC. Sensitivity analyses were performed using inverse probability weighting (IPW).^24^ Further information on the IPW analyses is presented in Supplement 3 (available online).

## Results

Means and variances for observed repeated measures of typical alcohol consumption and ASB for male and female participants are listed in Table S1 (available online).

### Parallel Growth Model for Typical Alcohol Consumption and ASB

**Figure 3:**
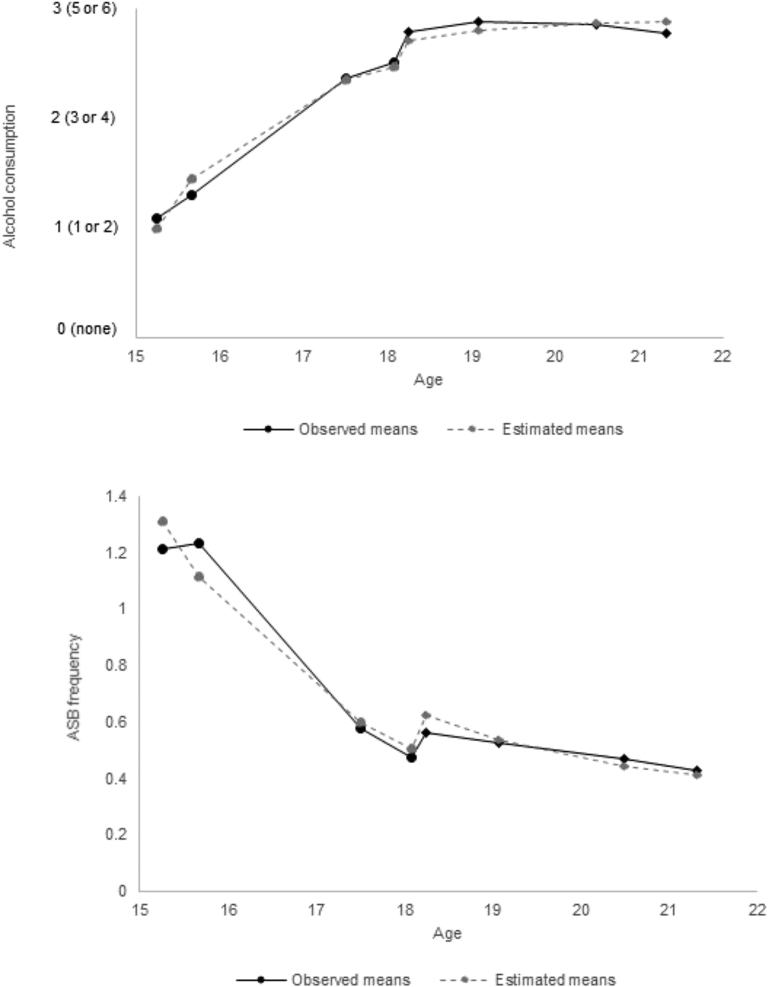
Observed and estimated means for units of typical alcohol consumption (exponential growth model) and frequency of antisocial behavior (ASB; exponential decay model; N = 6,112). Note: Circles = clinic assessments; diamonds = questionnaire assessments.

Estimated and observed means for the parallel growth model are shown in Figure 3, with means, variances, and correlations between growth factors presented in Supplement 4 and Table S2 (available online). The alcohol trajectory started at an average of 1.2 (SE 0.04) at 15 years 3 months of age (a score of 1 is equivalent to drinking “1 or 2” units of alcohol on a typical day when drinking) and increased to an average of 2.9 (SE 0.03; a score of 3 is equivalent to drinking “5 or 6” units of alcohol on a typical day when drinking). The factor loading for the final repeated alcohol measure on the asymptote suggested that 98% of the total change to the highest level was gained by the end of the study (21 years 4 months of age). The mean half-life for alcohol was 11 months (SE 0.06), indicating that, on average, young people reach a halfway point between their initial and final level of alcohol consumption at 16 years 3 months of age.

The ASB trajectory started at an average of 1.5 (SE 0.05) reported antisocial acts per year at 15 years of age and decreased to an average of 0.3 (SE 0.04) antisocial acts per year. The factor loading for the final repeated ASB measure suggested that 94% of the total change to the lowest level was gained by the end of the study. The mean half-life for ASB was 1 year 6 months (SE 0.20), indicating that, on average, young people reach a halfway point between their initial and final level of ASB at 16 years 10 months of age. Supplement 4 (available online) presents a description of correlations between alcohol and ASB growth factors.

### Between-Person Effects

**Figure 4:**
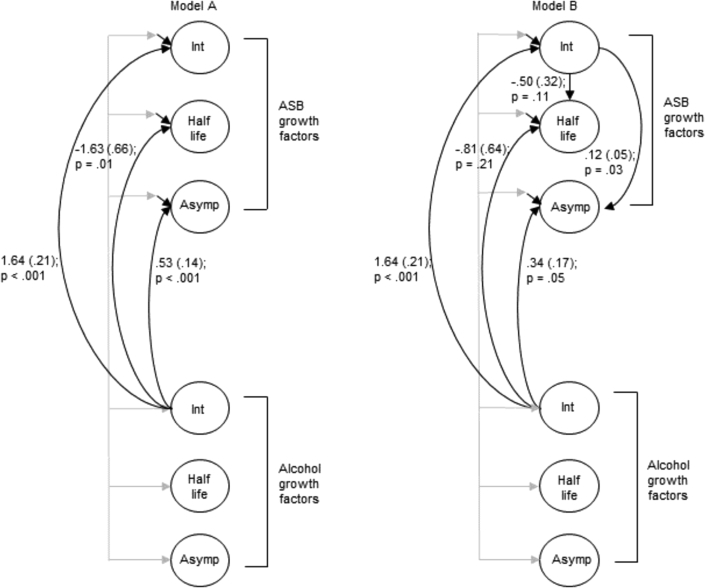
Between-person effects of typical alcohol consumption latent intercept on antisocial behavior (ASB) growth factors, showing unstandardized coefficient (standard error; N = 6,112). Note: Model A shows direct effects of alcohol intercept on ASB growth factors with residual covariances between ASB growth factors. Model B shows direct effects of alcohol intercept on ASB growth factors after taking account of the direct effect of ASB intercept on ASB half-life and asymptote. Asymp = asymptote; Int = Intercept.

Figure 4 shows that those with higher initial levels of alcohol consumption at 15 years of age also had higher initial levels of ASB (model A: b 1.64, SE 0.21; *p* < .001). In addition, those with higher initial levels of alcohol consumption had higher final levels of ASB (model A: b 0.53, SE 0.14; *p* < .001); however, this association weakened slightly when accounting for initial ASB levels (model B: b 0.34, SE 0.17; *p* = .05). There was a negative association between the alcohol intercept and the ASB half-life (model A: b −1.63, SE 0.66; *p* = .01), indicating that those with higher initial levels of drinking approached their final level of ASB more quickly; however, after adjustment for initial ASB levels, there was insufficient evidence of a relation between the alcohol intercept and ASB half-life (model B: b −0.81, SE 0.64; *p* = .21). All models included sociodemographic variables to address potential confounding and selection bias. Unadjusted results were very similar and therefore not shown.

### Within-Person Effects

There was evidence that within-person increases in alcohol consumption were associated with within-person increases in ASB from 18 to 21 years of age (b 0.06, SE 0.02; *p* = .001) but not from 15 to 18 years of age (b −0.02, SE 0.03; *p* = .48). These results indicate that, in early adulthood, time-specific increases in alcohol consumption relative to an individual’s own alcohol trajectory are associated with time-specific increases in ASB relative to an individual’s own ASB trajectory. Within- and between-person effects are presented in Table 1. Model fit statistics indicated a good fit to the data (root mean square error of approximation 0.02; comparative fit index 0.92).

**Table 1:** Between-Person Effects of the Alcohol Intercept on Antisocial Behavior (ASB) Growth Factors and Within-Person Effects of Repeated Alcohol Consumption on Repeated ASBs Across Different Sensitivity Analyses

	Original Model		Sensitivity Test 1		Sensitivity Test 2		Sensitivity Test 3		Sensitivity Test 4	
	Unstandardized Coefficient (SE)	*p* Value	Unstandardized Coefficient (SE)	*p* Value	Unstandardized Coefficient (SE)	*p* Value	Unstandardized Coefficient (SE)	*p* Value	Unstandardized Coefficient (SE)	*p* Value
Between-person effects of alcohol consumption intercept										
ASB intercept	1.64 (0.21)	<.001	1.55 (0.22)	<.001	1.72 (0.23)	<.001	0.91 (0.11)	<.001	1.94 (0.16)	<.001
ASB half-life	−0.81 (0.64)	.21	−0.79 (0.67)	.24	−0.89 (0.67)	.19	−0.95 (0.55)	.09	0.58 (0.57)	.30
ASB asymptote	0.34 (0.17)	.05	0.35 (0.18)	.05	0.34 (0.19)	.08	0.26 (0.11)	.02	0.17 (0.13)	.21
Within-person effects of repeated alcohol on repeated ASB										
Age 15–18 y^a^	−0.02 (0.03)	.48	−0.03 (0.04)	.37	−0.03 (0.04)	.45	−0.02 (0.02)	.23	0.13 (0.09)	.12
Age 18–21 y^b^	0.06 (0.02)	.001	0.06 (0.02)	.001	0.06 (0.02)	.001	0.04 (0.01)	.001	0.05 (0.04)	.28

Note: All results were adjusted for sex, housing tenure, maternal education, parity, and household crowding. “Original model” shows estimates from the final analysis model (model shown in Figure 2); “sensitivity test 1” shows estimates after also adjusting for childhood conduct problems, antisocial peers, and parental crime and alcoholism (n = 4,465 for these analyses); “sensitivity test 2” shows weighted estimates (from inverse probability weighting analyses); “sensitivity test 3” shows estimates using the total number of types of crime committed in the past year instead of frequency of crimes committed; “sensitivity test 4” shows estimates using alcohol frequency instead of typical consumption. SE = standard error.

a Parameter for clinic measurements constrained to equality.

b Parameter for questionnaire measurements constrained to equality.

### Sensitivity Analyses

The final model (Figure 2) was rerun to perform a number of sensitivity checks. First, analyses were adjusted for childhood conduct problems, exposure to antisocial peers, and parental crime and problematic alcohol use in addition to sociodemographic factors (Table 1; sensitivity test 1). Second, the impact of missing data was further assessed by using IPW (Table 1; sensitivity test 2). Third, the model was rerun using the total number of types of crime committed in the past year (range 0–8) instead of frequency (Table 1; sensitivity test 3). Fourth, the model was rerun using alcohol frequency instead of typical consumption (Table 1; sensitivity test 4). As presented in Table 1, conclusions were similar across all sensitivity analyses, with the exception of weaker evidence for within-person effects of frequency of alcohol consumption on ASB.

Fifth, although the theoretical justification and a priori hypothesis for this study were that excessive alcohol consumption affects ASB, it is possible that effects are present in the opposite direction. Therefore, the model was re-parameterized to examine the effect of the ASB intercept on the alcohol growth factors, after accounting for the effect of the alcohol intercept on the alcohol half-life and asymptote (Figure S4, available online). Those with higher initial ASB levels also had higher initial levels of alcohol consumption (b 0.19, SE 0.02; *p* < .001) but not final levels of alcohol consumption (b −0.04, SE 0.04; *p* = .31).

## Discussion

In this UK population-based sample, there was evidence for between- and within-person effects of alcohol consumption acting against desistance from ASB. That is, those who reported higher alcohol use compared with their peers in mid-adolescence also reported higher levels of ASB cross-sectionally and in early adulthood, although excessive alcohol use at the start of the study did not appear to affect the rate of decrease in ASB across adolescence. In addition, within those periods when young people reported consuming more alcohol than normal, they also reported engaging in more antisocial activities than would be expected given their overall pattern of ASB throughout adolescence and young adulthood. These time-specific effects were present in young adulthood but not in adolescence.

The results need to be interpreted in the context of several limitations. First, as with most cohort studies, there was selective attrition over time. Comparatively few cohort members provided data on all measures across adolescence and early adulthood. However, all analyses were performed using full information maximum likelihood estimation, which allowed more than 6,000 participants to be included, and the inclusion of auxiliary variables related to missing data or using IPW made little difference to the results. Second, typical alcohol consumption and ASB were assessed using questionnaires completed by respondents at home and computer-based sessions during focus clinics. To address any artifactual differences across assessment techniques, we incorporated this information into the derivation of the trajectories; however, the possibility remains that the lack of within-person effect of alcohol on ASB during adolescence is related to the data during this period being collected by a computer-based session rather than a postal survey.

Third, it is possible that the between-person association between alcohol use and ASB is spurious with common risk factors not examined in this study (such as shared genetic risk) causing alcohol use and ASB.^25^ However, accounting for earlier behavioral problems, exposure to antisocial peers and parental crime and problematic alcohol use had little impact on the between-person effects. Fourth, the effect size found for the within-person effects in early adulthood was small. However, this is because each individual serves as that individual’s control; therefore, all time-stable confounds that affect analyses examining between-person effects are eliminated^6^; in addition, within-person effects are the associations that remain even after accounting for the underlying growth trajectories for alcohol and ASB. Fifth, given the complexity of the models, there was insufficient power to examine differences in effect between men and women separately. However, previous research using the same sample has shown little evidence that associations between alcohol and ASB differ by gender.^26^, ^27^ In addition, the ASB items assessed in this study are rather “male-centric” and, in general, will capture only quite overt or confrontational behavior, although the prevalence of shoplifting and being rowdy was similar in male and female participants.

In the present study, we simultaneously estimated the between- and within-person effects of alcohol on ASB across adolescence and early adulthood using a population-based sample. Recent studies have begun to use similar techniques to tease apart the effects of heavy alcohol (or substance) use on dating aggression,^16^ psychopathic features,^17^ and crime levels in young adults in substance use treatment.^11^ The importance of disaggregating these effects has been highlighted to provide a more comprehensive investigation of the hypothesized developmental processes underlying the relation between behaviors that change together over time.^14^ Between-person effects tell us who is at risk, whereas within-person effects tell us when a person is at risk; therefore, these 2 effects provide unique information about the etiology of the association between alcohol and ASB and have important but different implications for theory and clinical practice.^12^, ^13^, ^14^

Consistent with previous research,^6^, ^7^, ^8^, ^11^, ^17^ between-person effects of baseline alcohol use on the course of ASB were identified, with higher typical alcohol consumption in mid-adolescence increasing initial and final levels of ASB. There also was a counterintuitive negative association between the alcohol intercept and the ASB half-life, indicating that higher alcohol consumption is associated with a faster decrease in ASB; however, this association disappeared after accounting for initial ASB levels. The negative association between baseline alcohol use and change in ASB over time has been reported previously, when initial levels of ASB are not taken into account.^6^, ^8^ These findings highlight the importance of exploring this association in greater detail by considering the effect of initial levels of ASB^9^ and investigating the effects of alcohol use on final levels of ASB, in addition to the rate of decrease.^6^ The ability to estimate the effect of alcohol use in mid-adolescence on the long-term course of ASB into adulthood is a key strength of this study. Using an exponential growth model not only allowed more complex and theoretically relevant longitudinal change to be examined but also enabled estimation of growth parameters that were of greater interest for research questions focused on ASB desistance (for further detail, see Supplement 2, available online). Although typical alcohol consumption in mid-adolescence had no effect on the rate of decrease of ASB, it was associated with final ASB levels in adulthood, even after accounting for ASB in mid-adolescence, indicating that excessive early alcohol use does have a long-term effect on ASB desistance.

The within-person effects identified in the present study were specific to young adulthood and were not present during adolescence. This finding supports previous research that has identified within-person effects across young adulthood.^6^, ^7^, ^8^, ^17^ Previous findings regarding within-person effects in adolescence have been inconsistent, with one study finding stronger effects of alcohol on dating aggression in adolescence compared with early adulthood^16^ and another finding no within-person effect of alcohol use on conduct problems in a sample of young adolescent girls.^15^ These conflicting findings are important to consider in the context of national variability in legal alcohol use. Early alcohol consumption might have a stronger relation to ASB in countries where it is not as normative, for example, in the United States, where the legal age for drinking is 21 years as opposed to 18 years in the United Kingdom. Within-person effects also were specific to the quantity of alcohol consumed rather than frequency, which supports previous research showing stronger evidence that quantity of alcohol consumed has a causal effect on violence.^28^

The present findings add to the literature on factors associated with desistance from ASB, highlighting that alcohol use can determine who is at risk and when they are at risk. Therefore, interventions should consider that the effects of alcohol on ASB are multifaceted. Future research should further investigate this relation by investigating pathways into excessive alcohol use and ASB in adolescence.
