# Supplementary material for: Effects of Excessive Alcohol Use on Antisocial Behavior Across Adolescence and Early Adulthood
Source: J Am Acad Child Adolesc Psychiatry. 2017 Oct;56(10):857–65. doi: 10.1016/j.jaac.2017.07.781 (PMC5625031; doi:10.1016/j.jaac.2017.07.781)
Supplement: Supplementary Data [file mmc1.docx]

**Supplement 1.** Detail on the Assessment of Confounders Used in Secondary Analyses

*Parental Crime and Alcohol Use*

Both were measured on eight occasions with questionnaires sent to mothers and their partners asking whether either had occurred since the last assessment. Any positive endorsement from either parent of being in trouble with the law/convicted from the child’s birth to 11 years was coded as positive for parental crime. Similarly, any report of alcoholism/alcohol problems across the same time period was coded as positive for parental problematic alcohol use.

*Childhood Conduct Problems*

Developmental trajectories of conduct problems between age 4 and 13 years have been derived previously.^1^ Briefly, latent class growth analysis models were applied to six binary indicators of conduct problems derived from the conduct problem scale of the Strengths and Difficulties Questionnaire,^2,3^ which was dichotomized at the threshold of 4 or more.^2^ The four resulting trajectories were described as low (64%), childhood-limited (15%), adolescent-onset (12%), and early-onset persistent (9%). Due to the complexity of the analysis model, the conduct problem latent classes were treated as an observed categorical covariate in analyses.

*Exposure to Antisocial Peers*

During a focus clinic at age ~11 years, children were asked whether any friends had committed a total of eleven antisocial acts (including smoking, alcohol use, cannabis use, stolen something, damaged property, assault, arson, carried a weapon, truancy, hurt animal, in trouble with police). All items were combined to create a sum score representing exposure to antisocial peers (range 0-11).

**Supplement 2.** Detail on the Derivation of the Parallel Exponential Growth Model

For constructs where non-linear change over time is hypothesized (as for antisocial behaviour [ASB] and alcohol use across adolescence and early adulthood), the most commonly used strategy is to add a quadratic term to the model. A trajectory model with a quadratic term (or additional higher-order polynomial functions) is non-linear with respect to time (change over time does not follow a linear pattern); however, the target function is linear with respect to the parameters and random coefficients, meaning that the model is straightforward to specify in a structural equation modeling framework.^4^ Specifically, the predicted score for a given individual at a particular time is simply a weighted sum of their individual intercept, slope, and quadratic scores.^5^

However, for behaviors such as ASB and alcohol use, a quadratic model is not consistent with underlying theory, given that, for example, ASB decreases between adolescence and adulthood, and this decrease slows with time until it begins to level off in early adulthood. Exponential models of change are being increasingly utilised within fields related to education and learning^4,6-10^; however, exponential change is also relevant for certain mental health domains. Although quadratic and exponential models may show similar patterns of change over short periods of time, over longer time periods, the quadratic curve will change direction and head toward positive/negative infinity, whereas the exponential curve will begin to level off and reach a plateau, a pattern of change that is much more realistic for many constructs including ASB and alcohol use. This plateau (the asymptote) can be estimated as part of the model and so allows the final levels of the construct of interest to be examined, alongside the initial levels (the intercept). Therefore, the exponential growth model not only allows more complex and theoretically relevant longitudinal change to be examined, but also enables estimation of growth parameters that are of greater interest for research questions focused on ASB desistance.

There are many variations on the equation describing exponential growth or decay:

| $y=\theta_{a}-\left( \theta_{i}-\theta_{a} \right)exp(-\theta_{r}t)$ | (1) |
| --- | --- |

Here $\theta_{i}$ represents the starting value (intercept), $\theta_{a}$ represents the horizontal asymptote, $\theta_{r}$ governs the rate of change, and $t$ represents time in years. Time was “centered” to start at zero to yield a more interpretative intercept mean (i.e., so the intercept mean represents average levels of ASB and alcohol at baseline [age 15 years] instead of at age 0). Here we opt to substitute the rate of change ($\theta_{r}$) for the half-life ($\theta_{h})$as proposed by a number of authors including Preacher and Hancock (2015) and Rausch (2004), although unlike previous studies, we combine this with the intercept and asymptote, as both are of substantive interest in the current study.

| $y=\theta_{a}-\left( \theta_{i}-\theta_{a} \right)\left( \frac{1}{2} \right)^{\frac{t}{\theta_{h}}}$ | (2) |
| --- | --- |

The exponential model is nonlinear in the sense of representing change over time, but additionally, the parameters in the model are related in a nonlinear way. When the target function is nonlinear with respect to the random coefficients, Taylor Series Expansion is needed (for further detail on Taylor Series Expansion see ^4,5,8^). Briefly, the partial derivative of the target function with respect to each growth parameter was taken so that the model could be expressed as a linear combination of latent variables. The partial derivatives with respect to each parameter of interest were:

$$\frac{\partial y}{\partial\theta_{i}}=-\left( \frac{1}{2} \right)^{\frac{t}{\theta_{h}}}$$

$$\frac{\partial y}{\partial\theta_{a}}=1-\left( \frac{1}{2} \right)^{\frac{t}{\theta_{h}}}$$

$$\frac{\partial y}{\partial\theta_{h}}=\left( \left( \frac{t}{{\theta_{h}}^{2}} \right)\left( \ln\left( \frac{1}{2} \right) \right)\left( \theta_{i}-\theta_{a} \right)\left( \left( \frac{1}{2} \right)^{\frac{t}{\theta_{h}}} \right) \right)$$

The linearized target function, shown below, was then specifiable using SEM.

$\tilde{y}=\mu_{a}-\left( \mu_{i}-\mu_{a} \right)\left( \frac{1}{2} \right)^{\frac{t}{\mu_{h}}}$ + ($\theta_{i}- \mu_{i})\left( -\left( \frac{1}{2} \right)^{\frac{t}{\mu_{h}}} \right)$ + ($\theta_{a}- \mu_{a})\left( 1-\left( \frac{1}{2} \right)^{\frac{t}{\mu_{h}}} \right)$

+ ($\theta_{h}- \mu_{h}) \left( \left( \frac{t}{{\mu_{h}}^{2}} \right)\left( \ln\left( \frac{1}{2} \right) \right)\left( \mu_{i}-\mu_{a} \right)\left( \left( \frac{1}{2} \right)^{\frac{t}{\mu_{h}}} \right) \right)$

The parallel exponential growth model was specified using a modified version of the SEM-based structured latent curve modelling (SLCM) approach^10,11^ for modelling nonlinear trajectories.^8^ This method has been described in detail elsewhere^8^; briefly, the means of the growth factors (intercept, half-life, and asymptote) were set to zero, and the intercepts of the repeated measures were constrained to equal the target function, in order to capture the mean trend. This approach was necessary in the current study to allow growth factors for ASB to be regressed directly on the latent intercept for alcohol consumption (to examine between-person effects). When using Taylor Series Approximation, predictors of random effects must be centered (with a mean of zero) to ensure that growth factors that enter the model nonlinearly (i.e., the half-life) maintain a mean of zero^4,5^; therefore, it was necessary for the alcohol latent intercept to have a mean of zero in the models estimating the between-person effect on the ASB growth factors.

**Supplement 3.** Detail on the Inverse Probability Weighting (IPW) Used to Address Missing Data

IPW has been recommended over alternative methods for dealing with missing data (such as multiple imputation) in situations where whole blocks of data are missing for a large proportion of individuals.^12^ In the ALSPAC sample, a large proportion of the young people (approximately 40%) provide no information on their alcohol use at any time. Weights were derived from a logistic regression analysis between a set of measures assessed in pregnancy that were independently predictive of missing data and/or variables in the analysis (maternal smoking in pregnancy, maternal alcohol use, maternal past severe depression, marital status, parity, child birthweight, and child gender) and inclusion in the final sample (*N* = 6,112/11,015). Minimal missing data on indicators used to derive weights were singly imputed as the modal or mean value (all indicators had < 8% of values missing). The Hosmer-Lemeshow test was used to assess the fit of the missingness model, with results showing no indication of poor fit (Hosmer-Lemeshow χ^2^ [df] = 12.72 [8]; *p* = .12). Weights ranged from 1.3 to 5.0.

**Supplement 4.** Means, Variances, and Correlations for ASB and Alcohol Growth Factors

There was a negative correlation between the intercept and half-life for alcohol consumption, indicating that those that had higher initial levels of alcohol use approached their final level of drinking more quickly (*r*[SE] = -0.27 [0.12], *p* = .03). Additionally, those that had higher initial levels of alcohol consumption had higher final levels (correlation between intercept and asymptote: 0.39 [0.06]; *p* < .001). Those who approached their final level of drinking more slowly, also had higher final levels (correlation between half-life and asymptote: 0.42 [0.05]; *p* < .001).

There was a negative correlation between the intercept and half-life for ASB, indicating that those who had higher initial levels of ASB approached their final level of ASB more quickly (*r*[SE] = -0.41 [0.13], *p* = .001). Additionally, those who had higher initial levels of ASB had higher final levels (correlation between intercept and asymptote: 0.68 [0.26]; *p =* .01).

There was a positive correlation between the alcohol intercept and both the ASB intercept and asymptote, indicating that those that had higher initial levels of drinking had higher initial levels (*r*[SE] = 0.54 [0.05], *p* < .001) and final levels of ASB (*r*[SE] = 0.61 [0.26], *p* = .02). Additionally, those that had higher initial levels of drinking approached their final level of ASB more quickly (*r*[SE] = -0.35 [0.12], *p* = .004). Those that had higher initial levels of ASB had higher final levels of alcohol (*r*[SE] = 0.18 [0.04], *p* = .002). There was also a weak negative correlation between the ASB intercept and alcohol half-life, indicating that those with higher initial levels of ASB approached their final level of drinking more quickly (*r*[SE] = -0.12 [0.07], *p* = .06). Finally, both half-lives were negatively correlated, indicating that those who approached their final level of drinking more quickly, approached their final level of ASB more slowly (*r*[SE] = -0.32 [0.16], *p* = .05), and both asymptotes were positively correlated, indicating that those that had higher final levels of drinking had higher final levels of ASB (*r*[SE] = 0.29 [0.13], *p* = .03).

**Table S1**. Means and Variances for Observed Repeated Measures of Typical Alcohol Consumption and Antisocial Behaviour (ASB) in Males and Females

| Age (years) | 15.3y | 15.7y | 17.5y | 18.1y | 18.3y | 19.1y | 20.5y | 21.3y |
| --- | --- | --- | --- | --- | --- | --- | --- | --- |
| Alcohol use (males) |  |  |  |  |  |  |  |  |
| Mean | 1.05 (0.04) | 1.25 (0.04) | 2.32 (0.05) | 2.58 (0.05) | 2.71 (0.07) | 2.87 (0.07) | 2.84 (0.06) | 2.85 (0.06) |
| Variance | 1.51 (0.09) | 1.91 (0.10) | 1.94 (0.08) | 1.93 (0.07) | 2.40 (0.10) | 2.35 (0.09) | 2.31 (0.08) | 2.16 (0.08) |
| Alcohol use (females) |  |  |  |  |  |  |  |  |
| Mean | 1.12 (0.04) | 1.35 (0.04) | 2.35 (0.04) | 2.43 (0.04) | 2.69 (0.05) | 2.77 (0.05) | 2.79 (0.04) | 2.70 (0.04) |
| Variance | 1.48 (0.08) | 1.78 (0.08) | 1.72 (0.07) | 1.69 (0.06) | 2.12 (0.07) | 2.11 (0.07) | 2.07 (0.06) | 2.13 (0.06) |
| ASB (males) |  |  |  |  |  |  |  |  |
| Mean | 1.61 (0.08) | 1.68 (0.08) | 0.73 (0.06) | 0.66 (0.05) | 0.66 (0.06) | 0.59 (0.06) | 0.64 (0.05) | 0.60 (0.04) |
| Variance | 7.21 (0.60) | 6.36 (0.51) | 2.54 (0.36) | 2.16 (0.27) | 2.07 (0.35) | 1.72 (0.34) | 1.63 (0.20) | 1.39 (0.16) |
| ASB (females) |  |  |  |  |  |  |  |  |
| Mean | 0.86 (0.05) | 0.85 (0.05) | 0.35 (0.03) | 0.26 (0.03) | 0.34 (0.03) | 0.39 (0.04) | 0.27 (0.03) | 0.27 (0.02) |
| Variance | 2.89 (0.30) | 2.73 (0.26) | 0.94 (0.12) | 0.71 (0.17) | 1.01 (0.16) | 1.23 (0.24) | 0.68 (0.13) | 0.61 (0.08) |

Note: Parameter estimate (standard error) shown; *n* = 2,772 for males and 3,340 for females.

**Table S2.** Means, Variances, and Correlations Between Antisocial Behavior (ASB) and Typical Alcohol Consumption (ALC) Growth Factors

|  | 1. | 2. | 3. | 4. | 5. | 6. |
| --- | --- | --- | --- | --- | --- | --- |
| 1. ALC intercept | 1 |  |  |  |  |  |
| 2. ALC half-life | -0.27 (0.12) | 1 |  |  |  |  |
| 3. ALC asymptote | 0.39 (0.06) | 0.42 (0.05) | 1 |  |  |  |
| 4. ASB intercept | 0.54 (0.05) | -0.12 (0.07) | 0.18 (0.04) | 1 |  |  |
| 5. ASB half-life | -0.35 (0.12) | -0.32 (0.16) | -0.11 (0.08) | -0.41 (0.13) | 1 |  |
| 6. ASB asymptote | 0.61 (0.26) | 0.09 (0.20) | 0.29 (0.13) | 0.68 (0.26) | -0.33 (0.28) | 1 |
| Mean | 1.22 (0.04) | 0.95 (0.06) | 2.89 (0.03) | 1.45 (0.05) | 1.52 (0.20) | 0.34 (0.04) |
| Variance | 0.61 (0.08) | 1.65 (0.38) | 1.28 (0.08) | 5.32 (0.32) | 10.90 (5.92) | 0.40 (0.25) |

Note: N = 6,112. Parameter estimate (standard error) shown.

**Figure S1.** Timeline for data collection. Note: ASB = antisocial behavior.


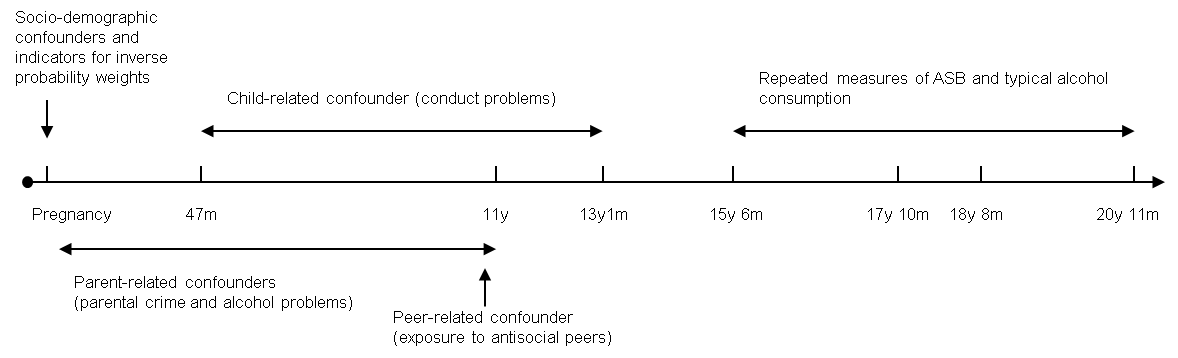


**Figure S2.** Comparison of between- and within-person analyses using an example exponential growth trajectory for alcohol use; Figure A shows between-person variability in the starting point (intercept) for alcohol use (the focus of between-person analyses); Figure B shows the time-specific residual at one time-point for individual A and B, e.g. the difference between what was observed and what would be predicted for that individual (the focus of within-person analyses).


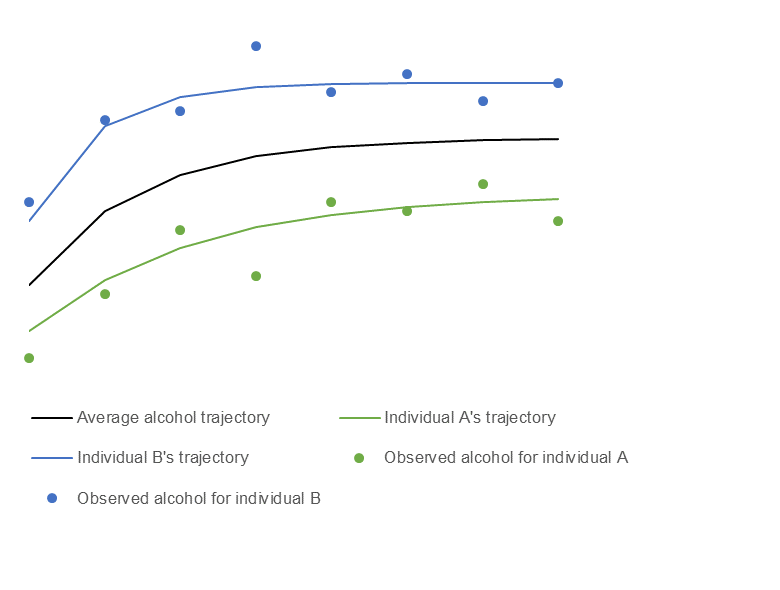


Between-person variation in the intercept


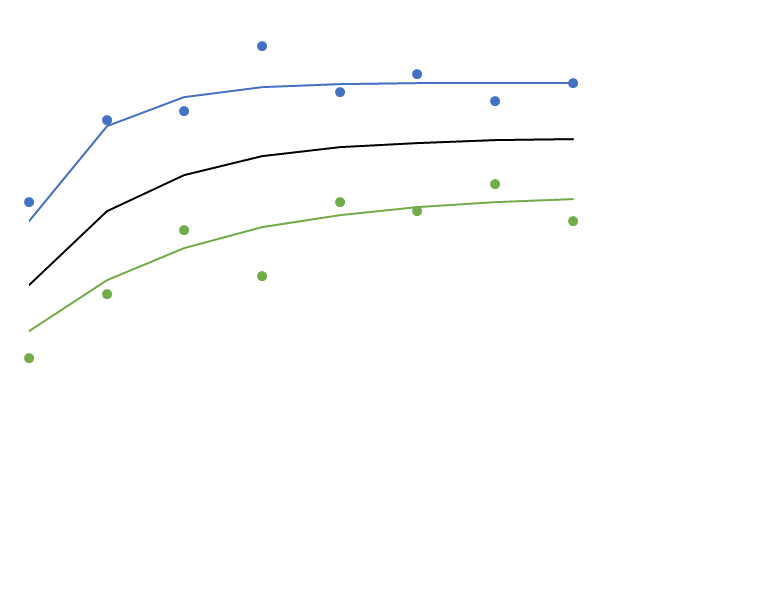


Time-specific residual

B

A

**Figure S3.** Flow chart of retention in the Avon Longitudinal Study of Parents and Children (ALSPAC) sample. Note: ASB = antisocial behaviour.

Complete data on socio-demographic confounders

N = 6,112

Data available for at least 1 of 4 repeated measures of ASB and alcohol consumption

n = 6,699

Sample used in main analyses

Sample used to derive inverse probability weights

Pregnant women enrolled in ALSPAC Phase 1

n = 14,541

Offspring alive at one year

n = 13,988

Initial sample after exclusions

n = 13,775

Invited to take part in at least 1 of 4 repeated assessments for ASB and alcohol consumption

n = 11,015

Exclusion criteria

Multiple births

Withdrawn from study

**Figure S4.** Between-person effects of antisocial behavior (ASB) latent intercept on alcohol consumption growth factors after taking account of the direct effect of alcohol intercept on alcohol half-life and asymptote. Note: N = 6,112. Figure shows unstandardized coefficient (standard error); Asymp = asymptote; Int = Intercept.

Int

Half life

Asymp

Int

Half life

Asymp

Alcohol growth factors

.19 (.02);

p < .001

.02 (.07);

p = .81

-.04 (.04);

p = .31

ASB

growth factors

-.43 (.25);

p = .09

.66 (.15);

p < .001

# **Supplementary references**

1. Barker ED, Maughan B. Differentiating early-onset persistent versus childhood-limited conduct problem youth. *Am J Psychiatry.* 2009;166:900–908.

2. Goodman R. Psychometric properties of the strengths and difficulties questionnaire. *J Am Acad Child Adolesc Psychiatry.* 2001;40:1337–1345.

3. Goodman R, Scott S. Comparing the strengths and difficulties questionnaire and the child behavior checklist: is small beautiful? *J Abnorm Child Psychol.* 1999;27:17–24.

4. Grimm K, Zhang Z, Hamagami F, Mazzocco M. Modeling nonlinear change via latent change and latent acceleration frameworks: Examining velocity and acceleration of growth trajectories. *Multivariate Behav Res*. 2013;48(1):117-143.

5. Grimm KJ, Ram N, Estabrook R. Nonlinear Structured Growth Mixture Models in Mplus and OpenMx. *Multivariate Behav Res*. 2011;45(6):887-909.

6. Blozis SA, Conger KJ, Harring JR. Nonlinear latent curve models for multivariate longitudinal data. *Int J Behav Dev*. 2007;31(4):340-346.

7. Kelley K, Maxwell SE. Delineating the average rate of change in longitudinal models. *J Educ Behav Stat*. 2008;33(3):307-332.

8. Preacher KJ, Hancock GR. Meaningful aspects of change as novel random coefficients : A general method for reparameterizing longitudinal models. *Psychol Methods*. 2015;20(1):84-101.

9. Zhang Z, McArdle JJ, Nesselroade JR. Growth rate models: emphasizing growth rate analysis through growth curve modeling. *J Appl Stat*. 2012;39(6):1241-1262.

10. Browne MW. Structured latent curve models. In: Cuadras CM, Rao CR, eds. Multivariate analysis: Future directions 2. Amsterdam, Netherlands: Elsevier-North-Holland; 1993:171–197

11. Browne MW, du Toit SHC. Models for learning data. In: Collins LM, Horn JL, eds. Best Methods for the Analysis of Change. Washington, DC: American Psychological Association; 1991:47-68.

12. Seaman SR, White IR, Copas AJ, Li L. Combining Multiple Imputation and Inverse-Probability Weighting. *Biometrics*. 2012;68(1):129-137.
